# A retrospective review of general paediatric inpatient deaths over time

**DOI:** 10.1186/1753-6561-9-S7-A3

**Published:** 2015-10-27

**Authors:** Amanda Roth, Jeremy Friedman, Adam Rapoport, Kim Widger

**Affiliations:** 1 Royal College of Surgeons in Ireland, Dublin, Ireland; 2 The Hospital for Sick Children, Toronto, Canada

## Background

To retrospectively review changes in the circumstances of general paediatric inpatient deaths at a tertiary hospital over three different time periods.

## Methods

Data was retrospectively collected for all patients who died on the General Paediatric Ward (the Ward) or in the Paediatric Intensive Care Unit (PICU) at this tertiary hospital, in the years 1998, 2005, and 2012. Patients who died in the PICU were considered to have a “general paediatric diagnosis” if their underlying condition or acute diagnosis would have normally resulted in admission to a General Paediatric Ward. The data elements collected were related to: demographic information about the child, health services data, information about provision and orders related to CPR at time of death and the involvement of palliative care services.

## Results

85 inpatients met the inclusion criteria; 35 in 1998, 27 in 2005, and 23 in 2012. Differences in location of death were noted across the three time periods. 94.3% of general paediatric patients died in the Paediatric Intensive Care Unit (PICU) in 1998; 59% died in the PICU in 2005, and 69.6% died in the PICU in 2012. The chronological age at which these children died decreased over the three time periods, varying from a median age of death of 5.96 years in 1998, to 4.58 years in 2012. The proportion of patients with ‘no Cardiopulmonary Resuscitation’ (no CPR) orders at the time of death increased over the 14 year period from 31% in 1998 to 87% in 2012. Similarly, the proportion of patients with palliative care involvement increased from 8.6% in 1998 to 73.9% in 2012.

## Conclusions

The number of inpatient general paediatric deaths at this tertiary hospital has decreased from 1998 to 2012. A larger proportion of these deaths are occurring on the Wards rather than in the PICU over time. ‘No CPR’ orders and palliative care consultations are becoming more prevalent in these patients prior to death.
